# Enhanced Killing of *Candida krusei* by Polymorphonuclear Leucocytes in the Presence of Subinhibitory Concentrations of *Melaleuca alternifolia* and “Mentha of Pancalieri” Essential Oils

**DOI:** 10.3390/molecules24213824

**Published:** 2019-10-23

**Authors:** Vivian Tullio, Janira Roana, Daniela Scalas, Narcisa Mandras

**Affiliations:** 1 Dept. Public Health and Pediatrics, Microbiology Division, University of Turin, 10126 Turin, Italy; janira.roana@unito.it (J.R.); narcisa.mandras@unito.it (N.M.); 2 Area Servizi Alla Ricerca Polo Agraria e Medicina Veterinaria, University of Turin, 10095 Turin, Italy; daniela.scalas@unito.it

**Keywords:** *Candida krusei*, PMNs, intracellular killing, essential oils, TTO, “Mentha of Pancalieri” EO

## Abstract

The aim of this study was to evaluate the influence of tea tree oil (TTO) and “Mentha of Pancalieri” essential oil (MPP) on intracellular killing of *Candida krusei,* often resistant to conventional drugs, by polymorphonuclear leucocytes (PMNs). Intracellular killing was investigated by incubating yeasts and PMNs with essential oils (EOs) at 1/4 and 1/8 × MIC (Minimal Inhibitory Concentration), in comparison with anidulafungin, used as a reference drug. Killing values were expressed as Survival Index (SI) values. The cytotoxicity of EOs was evaluated by 3-[4,-5-dimethylthiazole-2-yl]-2,5-diphenyltetrazolium bromide (MTT) assay. Both EOs were more efficaceous at 1/8 × MIC than 1/4 × MIC, with killing values higher than observed in EO-free systems and in presence of anidulafungin, indicating that the decreasing concentrations did not cause lower candidacidal activity. This better activity at 1/8 × MIC is probably due to the EOs’ toxicity at 1/4 × MIC, suggesting that at higher concentrations EOs might interfere with PMNs functionality. TTO and MPP at 1/8 × MIC significantly increased intracellular killing by PMNs through their direct action on the yeasts (both EOs) or on phagocytic cells (MPP), suggesting a positive interaction between EOs and PMNs to eradicate intracellular *C. krusei*. These data showed a promising potential application of TTO and “Mentha of Pancalieri” EO as natural adjuvants in *C. krusei* infection management.

## 1. Introduction

The growing threat of antimicrobial drug resistance to conventional antimicrobial drugs has stimulated the search for new therapeutic alternatives, including many extracts of medicinal plants and essential oils (EOs) that are now well recognized for their potential antimicrobial role against microorganisms [1,2]. It is estimated that at least 80% of the world population is still using traditional medicine, and plants represent a large source of bioactive compounds, which have resulted in the detection of a significant number of therapeutic properties [2,3,4]. However, the complex and diverse chemical structures of the EOs obtained from the medicinal plants require a more thorough investigation prior to their use as novel antimicrobial agents.

Clinical experience showed that the efficacy of conventional antimicrobial drugs depends both on their direct effect on microorganisms and on the activity of the host immune system to find compounds that are able to stimulate and not to interfere negatively with them [5,6,7]. To make a valid assessment of EOs activity for use in pharmaceutical applications and an effective comparison with conventional drugs, it is important to evaluate not only the classic microbiological parameters for assessing the antimicrobial efficacy but also the influence of the EOs on host defence mechanisms.

Hence, the purpose of this study was to evaluate the influence of the tea tree (*Melaleuca alternifolia*) oil (TTO) and the “Mentha of Pancalieri” (MPP) EO on intracellular killing by human polymorphonuclear leucocytes (PMNs), the first cell line of host defence, against *Candida krusei*, a yeast pathogen usual resistant to conventional drugs, in comparison with anidulafungin (AND), one of the antifungal drugs used in candidiasis management. PMNs are professional phagocytes, represent one of the effectors of innate immunity and play a primary role in the elimination of microbial pathogens against acute microbial infection. Consequently, the identification of substances, other than antibiotics, that can positively modulate these phagocytes is of great interest [7].

We chose these two EOs because of their interesting biological and microbiological properties, due to their richness in monoterpenes, a group of secondary metabolites of various family plants. Monoterpenes show many positive effects in humans, as they are able to diffuse into the bloodstream or to permeate the skin, acting as medicinal substances. The antitumor, antibacterial, antifungal, antiviral, immunomodulating, local anesthetic and anti-inflammatory properties of monoterpenes are promising. The EOs’ activity depends on the type of functional group of molecule, chemical structure, location of active moieties, solubility in fat, as well as the presence of aromatic nucleus. Moreover, the antifungal activity of EO components results from their interaction with enzymes linked to energy production and the synthesis of cell structural compound [8]. 

TTO is an EO obtained by steam distillation of the leaves and terminal branches of *Melaleuca alternifolia* [(Maiden & Betche) Cheel] (Myrtaceae). TTO was used early in last century as a topical agent for treating various diseases, predominantly dermatoses (e.g., recurrent herpes *labialis*, acne, pustules, dandruff, and rash) [9,10]. This efficacy in dermatology is probably due to the monoterpenes ability to permeate the skin thanks to their small molecular structure. One possible mechanism of action for this effect is the interaction of EOs and their component with skin lipids. Furthermore, different study concluded that the monoterpenes are suitable enhancers for skin permeation of lipophilic drugs [8]. TTO has a broad spectrum of antimicrobial activity against a wide range of bacteria, viruses, fungi, and parasites, as well as microorganisms that are resistant to conventional drugs [11,12,13,14]. In addition, TTO possesses anti-inflammatory properties, possibly due to its high concentration of terpinen-4-ol, a compound with anti-inflammatory activity [9].

MPP EO is a local variety of *Mentha x piperita* (Huds) [var. OFFICINALIS (Sole), form RUBESCENS (Camus) (Lamiaceae). from Pancalieri near Torino (Italy). In general, *M. piperita* L. (peppermint) EO has health benefits, and it is useful worldwide in gastrointestinal disorders, such as colics, dyspepsia, gastritis, etc. [15,16], and for chronic itching [17]. *M. piperita* L. possesses antibacterial and antifungal activity [18,19,20], but little is known of its potential ability to modulate the immune system, in particular the innate immune system. Recently, we detected that the EO of MPP has a good antifungal activity against yeasts both susceptible and resistant to azoles, including *Cryptococcus neoformans*, *C. krusei* and *C. glabrata*, and dermatophytes [21]. 

## 2. Results

The EOs used in this study were rich in monoterpenes. In particular, the main components of TTO were terpinen-4-ol (35.88%), γ-terpinene (19.65%), α-terpinene (8.64%), *p*-cymene (4.61%), and 1,8-cineole (4.07%), according to ISO data 4730:2004, while those of the MPP EO were menthol (41.7%) and menthone (21.8%), as previously reported, in accordance with data from the European Pharmacopoeia 8th Ed [21]. 

The MIC values of EOs together with the MIC values of AND are presented in Table 1. Comparison of MIC data demonstrated that both TTO and MPP EO displayed low MICs (% *v*/*v*) with a standard yeast inoculum of 10^3^ cfu/mL and possessed a significant fungicidal activity against *C.krusei*. In the presence of a higher yeast inoculum (10^6^ cfu/mL) necessary for carrying out the tests with phagocytes, the MICs obtained were 2–4 times higher (Table 1).

The patterns of intracellular killing toward *C. krusei* by PMNs in presence of EOs or AND are shown in Table 2. In all assays, the viability of EO/drug free PMNs remained unchanged during the incubation period for up to over 90 min. The results showed that control systems (EOs/drug free PMNs), were able to kill yeast cells only in part, with Survival Index (SI) values of 1.78-1.71-1.67 (corresponding to 22-29-33% killing), during the 90 min incubation period. 

**Figure 1:**
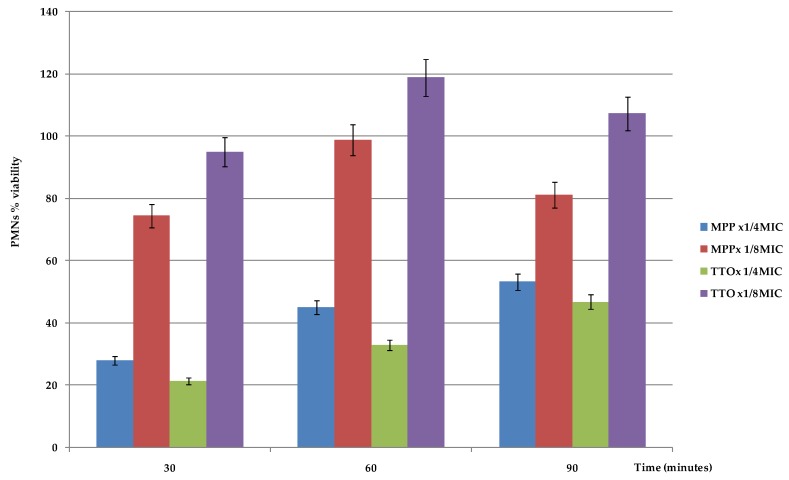
PMN percent survival: effect of sub-inhibitory concentrations of *Melaleuca alternifolia* (TTO) and “Mentha of Pancalieri” (MPP) essential oils on PMN viability at 30, 60, and 90 min. Data presented as the average ± SEM. TTO = Tea Tree Oil; MPP = “Mentha of Pancalieri” essential oil.

Conversely, TTO and, more markedly MPP, added to PMNs at sub-inhibiting concentrations, significantly enhanced phagocyte intracellular fungicidal activity against ingested yeasts (Table 2). The addition of 1/4xMIC of TTO or MPP produced a significantly decrease in the survival of yeasts, with SI values ranging from 1.50 to 1.60 and from 1.39 to 1.54 respectively (corresponding to killing values from 40 to 50% and 46 to 61%), during the entire incubation period, in comparison with controls (Sis = 1.78-1.71-1.67) (*p* < 0.01 and *p* < 0.05). In the systems containing EOs at lower concentrations (1/8 × MIC), there was a further decrease in the yeast survival, with SIs of 1.45-1.41-1.38 for TTO and 1.33-1.50-1.47 for MPP during the incubation period, respectively (*p* < 0.01 and *p* < 0.05; Table 2). These results are probably due to the fact that, as the MTT assay showed (Figure 1), both the EOs were found to be toxic to PMNs at the highest concentration of 1/4 × MIC, interfering with their functionality. In the presence of 1/2 × MIC AND intracellular yeasts were killed at similar values to the controls (Sis = 1.73-1.67-1.67correponding to killing values of 27-33-33%).

To investigate the direct effect of EOs and the drug on the intra-phagocyte killing, yeast cells and PMNs were pre-incubated for 60 min with sub-inhibiting concentrations of TTO, MPP, or AND. After withdrawal of the antimicrobial agents, the fungicidal activity was determined. 

Pre-treated yeasts with 1/4–1/8 × MIC MPP were killed more efficiently by PMNs than controls and AND pre-treated yeasts; in fact, SIs were 1.54-1.56-1.43 (46-44-57% killing) and 1.50-1.53-1.40 (50-47-60%) vs. 17-18-13% (1/2 × MIC AND) and 22-29-33% (yeasts not pre-treated) (*p* < 0.01 and *p* < 0.05; Table 3). Similarly, when yeast cells were pre-exposed to 1/4–1/8 × MIC TTO, killing activity by PMNs significantly increased compared to controls and yeasts pre-treated with AND, although at lower percentages than those observed with MPP (Table 3).

Pre-treatment of PMNs with 1/8 × MIC MPP resulted in a significant enhancement of intracellular killing of *C. krusei*, as compared with 1/2 × MIC AND and control systems (*p* < 0.01 and *p* < 0.05; Table 4). In contrast, pre-exposure of PMNs to 1/4–1/8 × MIC of TTO had no effect on intra-phagocyte killing of *C. krusei*, with SI values higher than controls (Table 4).

## 3. Discussion

During the last three decades, the number of fungal infections caused by yeasts of *Candida* spp. has increased dramatically, mainly due to the rise in the number of immunocompromised patients [22]. After the advent of HIV infection, the widespread use of fluconazole in these patients have also contributed to a significant increase in *C. krusei* infection, particularly because of its natural resistance to this azole and other antifungal drugs [23]. The possibility that OEs may have antifungal activity towards *C. krusei* and may be able to stimulate immune system functions becomes particularly important, especially from a therapeutic point of view [24,25,26,27]. However, since data on the effects of EOs on the innate immune system are contradictory and fragmentary, the mechanisms of EOs interactions with the innate immune system are not yet completely clear. 

The results obtained in this study with TTO and MPP emphasize that, in comparison with control systems and AND, the EOs, at sub-inhibiting concentrations (1/4 and 1/8 × MIC), produced a significantly higher intracellular killing by PMNs against *C. krusei* (*p* < 0.01 and *p* < 0.05; Table 2) throughout the entire incubation time. However, TTO and MPP at higher concentrations (1/4 × MIC) displayed killing values lower than those detected at 1/8 × MIC. These results are probably due to the fact that these EOs at higher concentrations are toxic for PMNs, as we detected by MTT assay (Figure 1), and could interfere with PMN functionality, reducing their ability to kill intracellular yeast cells [28]. A similar phenomenon was first observed for the antifungal drug caspofungin in *C. albicans* by Stevens et al. [29] and is known as the “paradoxical effect” or “eagle effect”. Further investigations are needed to clarify this mechanism for EOs.

It should be noted that both EOs, albeit at a different percentage, show the ability to stimulate the intracellular killing activity of PMNs, in comparison with controls and AND, even at 1/8 × MIC, indicating that the decreasing concentrations did not cause lower candidacidal activity.

To determine whether the increased killing activity of EOs was due to their direct action on yeast cells or PMNs or both, yeast cells or phagocytes were separately exposed to EOs for 60 min prior to killing tests. Yeast cells pre-treated with 1/4 or 1/8 × MIC of TTO or MPP were killed more efficiently by PMNs (*p* < 0.01; Table 3) than untreated *C. krusei*. These results might be related to some direct EOs-induced changes on the morphology and/or physiology of *C. krusei* that might indirectly affect its virulence, making yeast cells more susceptible to PMN lytic mechanisms. In fact, it is known that TTO and MPP are able to alter fungal cell wall and/or cellular membrane integrity so that the PMNs may react toward *C. krusei* in a more positive manner [30,31,32,33].

On the other hand, pre-exposure of PMNs to 1/4 × MIC of TTO did not enhance the intracellular killing of *C. krusei* (Table 4), because after the withdrawal of the EO, killing values were lower than those observed with the untreated controls, suggesting that TTO at 1/4 MIC affects PMNs functionality in eradicating intracellular yeast cells. PMNs pre-treated with 1/8 × MIC of TTO displayed a slightly decreased in intracellular killing, in comparison with untreated PMNS, indicating that TTO at this concentration did not adversely interfere with PMN functionality. The mechanism behind these results is not known but we speculate that pre-exposure to TTO could masks or changes some cellular receptors that prevent yeasts recognition and killing by PMNs, as we previously observed with caspofungin [6]. 

Conversely, pre-treatment of PMNs with 1/8 × MIC MPP resulted in a significant enhancement of intracellular killing of *C. krusei*, as compared with ½ MIC × AND and control systems (*p* < 0.01 and *p* < 0.05) (Table 4), showing that MPP is able to stimulate fungicidal activity by PMNs even at lower concentrations. 

These data are difficult to compare with other studies, because there are no available reports in the literature on the interaction of TTO and/or MPP, PMNs and *C. krusei*. In fact, most investigations on EOs and their influence on immune system are mainly focused on their antioxidant and anti-inflammatory properties, ROS production, or other phagocyte pathways [34,35,36,37,38]. However, our results are consistent with our previous data showing the ability of thyme red EO or antifungal agents to induce stimulation of phagocyte functions in eradicating intracellular *C. albicans* [6,27].

## 4. Materials and Methods 

### 4.1. Yeasts

A recent clinical *C. krusei* strain isolated from blood was used. The yeast was identified by the API ID32C system method (BioMérieux, Rome, Italy) and subcultured on Sabouraud dextrose agar (SDA; Oxoid S.p.A., Milan, Italy) at 35 °C before the experiments. Yeast cultures consisted entirely of blastoconidia and had a slight tendency to differentiate into pseudohyphae during the experiments [6,26].

### 4.2. Essential Oils 

The TTO (batch n. 140,208 year 2014) was purchased from Flora (Pisa, Italy), while MPP EO obtained from the fresh leaves of *M. piperita* (Huds) var. OFFICINALIS (Sole), form RUBESCENS (Camus) (Lamiaceae) by steam distillation, was purchased from Erbe Aromatiche Essenzialmenta, Pancalieri (Turin, Italy) [21]. TTO composition was determined by GC-MS at Flora s.r.l. with a Clarus 500 gas cromatograph (Perkin Elmer, Milan, Italy). MPP EO was analysed by GC-MS at Drug Science and Technology Department (University of Turin, Italy) as previously described [21].

For the experimental assays, EOs were dissolved in ethanol (1:2.5), and diluted (1:20) up to 2% (*v*/*v*) in RPMI-1640 medium with L-glutamine plus 0.2% glucose, and without sodium bicarbonate (Sigma-Aldrich, Rome, Italy), as previously described [21,39]. Then, EO solutions were buffered to pH = 7 with 0.165 M morpholinepropanesulfonic acid (MOPS) (Sigma-Aldrich). The EOs were protected from light and humidity and maintained at 4 °C just before use [21,39]. The final EO concentrations ranging from 1% to 0.0019% (*v*/*v*) and ethanol maximum concentration was 1.5% (*v*/*v*). Growth controls consisted of RPMI-1640 medium and RPMI-1640 contained 1.5% (*v*/*v*) ethanol [21,39].

### 4.3. Reference Antifungal Agent 

AND was kindly provided by Pfizer Italia (Rome, Italy). Stock solutions were prepared into pyrogen- free distilled water, and stored at −20 °C just before use [6]. 

### 4.4. In Vitro Antifungal Susceptibility Assays

*C. krusei* was tested for susceptibility to TTO, MPP EO, and to AND by a broth microdilution method, in accordance to CLSI guidelines (CLSI M27-A3 and M27-S4) [40,41], with some modifications for the EOs, as previously described [21]. 

EOs and AND MIC values for *C. krusei* were determined with an inoculum of 10^3^ cfu/mL and an inoculum of 10^6^ cfu/mL to perform tests with phagocytes [6,27]. 

MFC, determined by inoculating 10 μL from non-turbid wells on SDA agar plates and incubated for 72 h at 30 °C, was defined as the lowest concentration resulting in no growth on subculture [21,39].

### 4.5. Human Polymorphonuclear Leucocytes (PMNs)

PMNs were separated from lithium heparinized venous human blood using Ficoll–Paque (Pharmacia S.p.A., Milan, Italy), and adjusted to 10^6^ cells/mL in RPMI 1640 medium (Sigma-Aldrich) as previously described in detail [5,42]. PMN viability, determined by trypan blue exclusion, was greater than 95% [43].

### 4.6. Influence of Antimicrobial Agents on Intracellular Killing by PMNs

Intracellular killing of *C. krusei* by PMNs was investigated by incubating together blastoconidia (10^6^ cfu/mL) and PMNs (10^6^ cells/mL) (1:1 ratio) at 37 °C for 30 min to allow yeast cells are phagocytosed by phagocytes. The PMN: yeast cells mixtures were centrifuged at 200× *g* for 5 min and washed to remove extracellular blastoconidia [6,26]. After that, an aliquot of PMNs was lysed by adding sterile water, and intracellular viable yeast cell counting was performed (time zero) [6,26]. The EOs/AND effect on the intracellular killing was evaluated by incubating PMNs further with sub-inhibitory concentrations of EOs (1/4 and 1/8 MIC), and 1/2MIC of AND. EOs/AND-free controls were included. At time x (30, 60 and 90 min), the viable counts were measured in the above same way [6,26].

Killing values were extrapolated from the survival indexes (SIs), which were calculated by adding the number of surviving yeast cells at time zero to the number of survivors at time x, and dividing by the number of survivors at time zero. According to this formula, if fungal killing was 100% effective, the SI would be 1 [6,26].

To differentiate between any separate effect of EOs and AND on *C. krusei* and the PMNs, the experiments were conducted after exposure of each of them to sub-inhibiting EOs/AND concentrations for 1 h, before they were incubated together [5,6]. After the withdrawal of the EOs or AND, pre-exposed yeast cells were added to PMNs, and yeasts cells to pre-exposed PMNs. A control system was assayed in parallel with no EOs or AND. The fungicidal activity of PMNs was determined as described above [5,6]. 

### 4.7. PMNs Viability Evaluation in the Presence of EOs

The EOs cytotoxicity on PMNs was evaluated using the 3-(4,5-dimethylthiazol- 2-yl)-2,5diphenyltetrazolium bromide (MTT) assay. PMNs (10^6^ cells/mL) were seeded in 96 microtiter plates in RPMI 1640 and incubated at 37° for 30′, 60′, and 90′ with 20 μL of 5 mg/mL MTT in PBS and different concentrations of EOs. After plate centrifugation and cell supernatant discarding, the dark blue formazan crystals were dissolved using 100 μL of sodium dodecyl solphate (SDS). The plates were read on a Synergy HT microplate reader (BioTek Instruments, Winooski, VT, USA) at a test wavelength of 550 nm and at a reference wavelength of 650 nm. Data were expressed as percentage of PMN viability. Reduction of cell viability by more than 30% was considered a cytotoxic effect [44].

### 4.8. Statistical Analysis

Each test was performed in quadruplicate, and the results were compared with those obtained with the controls and expressed as the means and standard errors of the means (SEMs) for 10 separate experiments. Statistical evaluation of the differences between test and control results was performed by analysis of variance using Tukey’s test. A *p* value of <0.05 was considered significant.

## 5. Conclusions

Taken together the results of this study showed that either TTO or MPP are able to significantly increase intracellular killing by PMNs through their direct action on the yeasts (both EOs) or on phagocytic cells (MPP), suggesting a positive interaction between EOs and PMNs in eradicating intracellular *C. krusei*, and providing further data on the biological properties of these EOs. These data showed a promising potential application of TTO and MPP EO as natural adjuvant for management of infections by *C. krusei*, which is often resistant to the most common antifungal drugs. Although the in vitro results are promising, indicating good activity of these EOs even at low amounts, further researches on bioavailability, pharmacokinetics and tolerability together with pre-clinical and clinical investigations, are necessary to assess the in vivo efficacy of OE preparations.

## Figures and Tables

**Table 1:** Susceptibility of *Candida krusei* to tea tree Oil (TTO), “Mentha of Pancalieri” EO, anidulafungin (AND) expressed as Minimal Inhibitory Concentration (MIC) and Minimal Fungicidal Concentration (MFC).

Drug	10^3^ cfu/mL	10^6^ cfu/mL
***Tea tree oil (TTO)***		
MIC	0.5% *v*/*v*	1% *v*/*v*
MFC	0.5% *v*/*v*	1% *v*/*v*
***Mentha of Pancalieri EO***		
MIC	0.25% *v*/*v*	1% *v*/*v*
MFC	0.5% *v*/*v*	1% *v*/*v*
***Anidulafungin (AND)***		
MIC	1 μg/mL	8 μg/mL
MFC	16 μg/mL	>16 μg/mL

**Table 2:** Effect of sub-inhibitory concentrations of *Melaleuca alternifolia* (TTO) and “Mentha of Pancalieri” (MPP) essential oils in comparison with anidulafungin (AND) on intracellular killing of *Candida krusei* by human PMNs. The PMN killing values were expressed as a Survival Index ± SEM (standard errors of the means), calculated as the number of surviving microorganisms at 30 min (T_o_) plus the number of surviving microorganisms at T_x_ divided by the number of surviving microorganisms at T_o_.

Survival Index ± SEM						
Time (min)	Controls	AND 1/2 × MIC (4 μg/mL)	TTO 1/4 × MIC 0.25% (*v*/*v*)	TTO 1/8 × MIC 0.125% (*v*/*v*)	MPP 1/4 × MIC 0.25% (*v*/*v*)	MPP 1/8 × MIC 0.125% (*v*/*v*)
30	1.78 ± 0.16 (22%)^c^	1.73 ± 0.16 (27%)	1.50 ^a^ ± 0.06 (50%)	1.45 ^a^ ± 0.05 (55%)	1.39 ^a^ ± 0.08 (61%)	1.33 ^a^ ± 0.09 (67%)
60	1.71 ± 0.01 (29%)	1.67 ± 0.19 (33%)	1.60 ^b^ ± 0.14 (40%)	1.41 ^a^ ± 0.07 (59%)	1.54 ^b^ ± 0.02 (46%)	1.5 ^a^ ± 0.11 (49%)
90	1.67 ± 0.15 (33%)	1.67 ± 0.08 (33%)	1.58 ^b^ ± 0.01 (42%)	1.38 ^a^ ± 0.08 (62%)	1.44 ^a^ ± 0.01 (56%)	1.47 ^a^ ± 0.19(53%)

**^a^** Significantly different from the controls (*p* < 0.01); **^b^** Significantly different from the controls (*p* < 0.05). **^c^** % Percentages of initial fungal population killed by PMNs in absence/presence of the essential oils and drug. AND = anidulafungin; TTO = Tea Tree Oil; MPP = “Mentha of Pancalieri” essential oil.

**Table 3:** Effect of 1 h of pre-exposure of *Candida krusei* to anidulafungin (1/2 × MIC), TTO or “Mentha of Pancalieri” essential oil (1/4 × MIC; 1/8 × MIC) on PMNs intracellular killing. The PMN killing values were expressed as a Survival Index ± SEM (standard errors of the means), calculated as the number of surviving microorganisms at 30 min (T_o_) plus the number of surviving microorganisms at T_x_ divided by the number of surviving microorganisms at T_o_.

Survival Index ± SEM						
Time (min)	Controls	AND 1/2 × MIC (4 μg/mL)	TTO 1/4 × MIC 0.25% (*v*/*v*)	TTO 1/8 × MIC 0.125% (*v*/*v*)	MPP 1/4 × MIC 0.25% (*v*/*v*)	MPP 1/8 × MIC 0.125% (*v*/*v*)
30	1.78 ± 0.16 (22%)^c^	1.83 ± 0.06 (17%)	1.58 ^b^ ± 0.15 (42%)	1.55 ^a^ ± 0.01 (45%)	1.54 ^a^ ± 0.15 (46%)	1.50 ^a^ ± 0.01 (50%)
60	1.71 ± 0.01 (29%)	1.82 ± 0.06 (18%)	1.61 ± 0.14 (39%)	1.57 ^a^ ± 0.06 (43%)	1.56 ^b^ ± 0.14 (44%)	1.53 ^a^ ± 0.06(47%)
90	1.67 ± 0.15 (33%)	1.87 ± 0.03 (13%)	1.64 ± 0.04 (36%)	1.61 ^a^ ± 0.01 (39%)	1.43 ^a^ ± 0.04 (57%)	1.40 ^a^ ± 0.01(60%)

**^a^** Significantly different from the controls (*p* < 0.01); **^b^** Significantly different from the controls (*p* < 0.05). **^c^** % Percentages of initial fungal population killed by PMNs in absence/presence of the essential oils and drug. AND = anidulafungin; TTO = Tea Tree Oil; MPP = “Mentha of Pancalieri” essential oil.

**Table 4:** Effect of 1 h of pre-exposure of human PMNs to anidulafungin (1/2 × MIC) or TTO or “Mentha of Pancalieri” essential oil (1/4 × MIC; 1/8 × MIC) on PMNs intracellular killing of *Candida krusei*. The PMN killing values were expressed as a Survival Index ± SEM (standard errors of the means), calculated as the number of surviving microorganisms at 30 min (T_o_) plus the number of surviving microorganisms at T_x_ divided by the number of surviving microorganisms at T_o_.

Survival Index ± SEM						
Time (min)	Controls	AND 1/2 × MIC (4 μg/mL)	TTO 1/4 × MIC 0.25% (*v*/*v*)	TTO 1/8 × MIC 0.125% (*v*/*v*)	MPP 1/4 × MIC 0.25% (*v*/*v*)	MPP 1/8 × MIC 0.125% (*v*/*v*)
30	1.78 ± 0.16 (22%)^c^	1.52 ^b^ ± 0.13 (48%)	1.64 ± 0.05 (14%)	1.83 ± 0.06 (17%)	1.56 ^b^ ± 0.11 (44%)	1.36 ^a^ ± 0.03 (64%)
60	1.71 ± 0.01 (29%)	1.55 ^b^ ± 0.15 (45%)	1.88 ± 0.09 (12%)	1.78 ± 0.02 (22%)	1.70 ± 0.11 (30%)	1.46 ^a^ ± 0.14 (54%)
90	1.67 ± 0.15 (33%)	1.40 ^a^ ± 0.08 (60%)	1.88 ± 0.05 (12%)	1.76 ± 0.11 (24%)	1.83 ± 0.02 (27%)	1.39 ^a^ ± 0.13(61%)

**^a^** Significantly different from the controls (*p* < 0.01); **^b^** Significantly different from the controls (*p* < 0.05). **^c^** % Percentages of initial fungal population killed by PMNs in absence/presence of the essential oils and drug. AND = anidulafungin; TTO = Tea Tree Oil; MPP = “Mentha of Pancalieri” essential oil.
